# Four-layered [3.3]meta­cyclo­phane with ethene­tetra­carbo­nitrile

**DOI:** 10.1107/S1600536814009362

**Published:** 2014-04-30

**Authors:** Masahiko Shibahara, Motonori Watanabe, Kenta Goto, Teruo Shinmyozu

**Affiliations:** a Department of Chemistry, Faculty of Education and Welfare Science, Oita University, 700 Dannoharu, Oita 870-1192, Japan; b International Institute for Carbon-Neutral Energy Research (I2CNER), Kyushu University, 744 Motooka, Nishi-ku, Fukuoka 819-0395, Japan; c Institute for Materials Chemistry and Engineering (IMCE), Kyushu University, 6-10-1 Hakozaki, Higashi-ku, Fukuoka 812-8581, Japan

## Abstract

The title complex C_42_H_48_·2C_6_N_4_ {systematic name: hepta­cyclo[21.13.1.1^5,19^.1^6,18^.1^10,14^.1^24,36^.1^28,32^]do­tetra­conta-1(37),5(40),6(41),10(42),11,13,18,23,28,30,32(39),36(38)-dodeca­ene–ethene­tetra­carbo­nitrile (1/2)}, consisting of four-layered [3.3]meta­cyclo­phane (MCP) with two tetra­cyano­ethyl­ene (TCNE) mol­ecules, was grown from a mixture of MCP and TCNE in chloro­form solution. The four-layered [3.3]MCP has an S-shaped structure in which three [3.3]MCP moieties take *syn*-(chair-boat), *anti*-(chair-boat) and *syn*-(chair-boat) conformations. The two outer [3.3]MCP moieties with *syn* geometry contain benzene rings with a tilt of 32.95 (7)°. The central [3.3]MCP moiety has an *anti* geometry, in which the two benzene rings are oriented parallel to each other at a transannular distance of 2.31 Å. The TCNE mol­ecules are stacked on either side of the outer [3.3]MCP units at a distance of 3.19 Å on one side and 3.24 Å on the other, and showed 0.80:0.20 and 0.44:0.56 disorder, respectively.

## Related literature   

For the previously reported C_42_H_48_·C_6_N_4_ (1:1) complex, see: Shibahara *et al.* (2011*a* ▶). For the free ligand C_42_H_48_, see: Shibahara *et al.* (2007 ▶). For multilayered [3.3]para­cyclo­phanes, see: Shibahara *et al.* (2008 ▶, 2011*a* ▶,*b* ▶). For cyclo­phanes, see: Vögtle (1993 ▶).
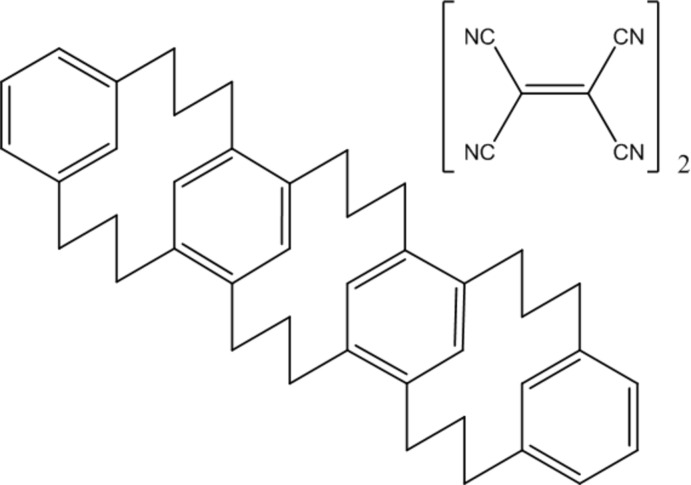


## Experimental   

### 

#### Crystal data  

C_42_H_48_·2C_6_N_4_
*M* *_r_* = 809.03
Triclinic, 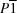

*a* = 9.563 (3) Å
*b* = 10.101 (4) Å
*c* = 11.679 (4) Å
α = 96.365 (14)°
β = 99.134 (13)°
γ = 107.683 (13)°
*V* = 1045.9 (6) Å^3^
*Z* = 1
Mo *K*α radiation
μ = 0.08 mm^−1^
*T* = 123 K
0.35 × 0.16 × 0.09 mm

#### Data collection  

Rigaku R-AXIS RAPID diffractometer
17470 measured reflections
4775 independent reflections
4141 reflections with *F* ^2^ > 2σ(*F* ^2^)
*R*_int_ = 0.050

#### Refinement  

*R*[*F* ^2^ > 2σ(*F* ^2^)] = 0.060
*wR*(*F* ^2^) = 0.178
*S* = 1.00
4775 reflections
394 parameters
Only H-atom coordinates refined
Δρ_max_ = 0.80 e Å^−3^
Δρ_min_ = −0.50 e Å^−3^

### 

Data collection: *RAPID-AUTO* (Rigaku, 1998 ▶); cell refinement: *RAPID-AUTO*; data reduction: *RAPID-AUTO*; program(s) used to solve structure: *SHELXD* (Schneider & Sheldrick, 2002 ▶); program(s) used to refine structure: *SHELXL97* (Sheldrick, 2008 ▶); molecular graphics: *CrystalStructure* (Rigaku, 2010 ▶); software used to prepare material for publication: *CrystalStructure*.

## Supplementary Material

Crystal structure: contains datablock(s) General, I. DOI: 10.1107/S1600536814009362/gw2145sup1.cif

Structure factors: contains datablock(s) I. DOI: 10.1107/S1600536814009362/gw2145Isup2.hkl

CCDC reference: 999480

Additional supporting information:  crystallographic information; 3D view; checkCIF report

## Figures and Tables

**Fig. 1.**
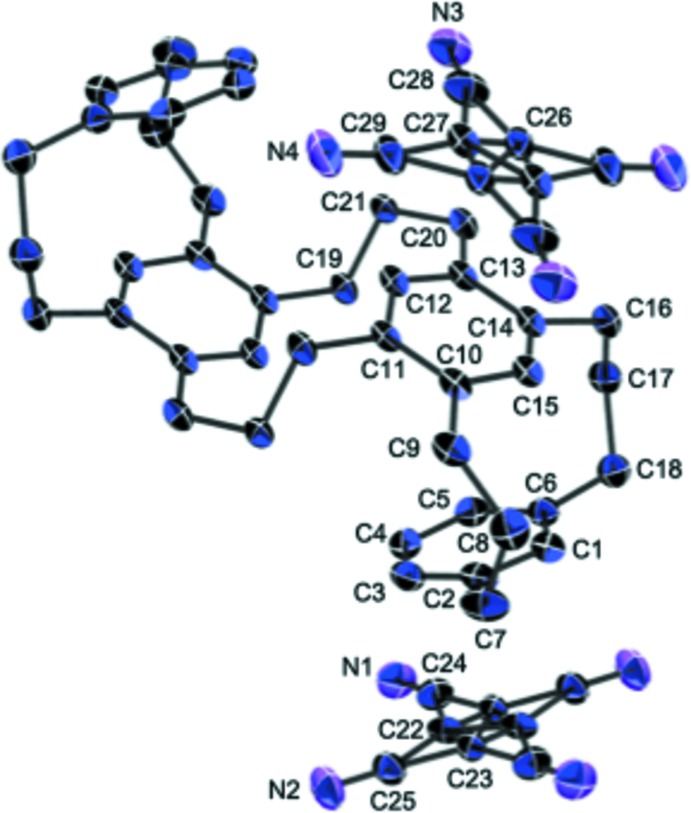
The molecular structure of C42H48.2C6N4 with the atom-labelling scheme and displacement ellipsoids are drawn at the 50% probability level. H atoms are omitted for clarity.

**Fig. 2.**
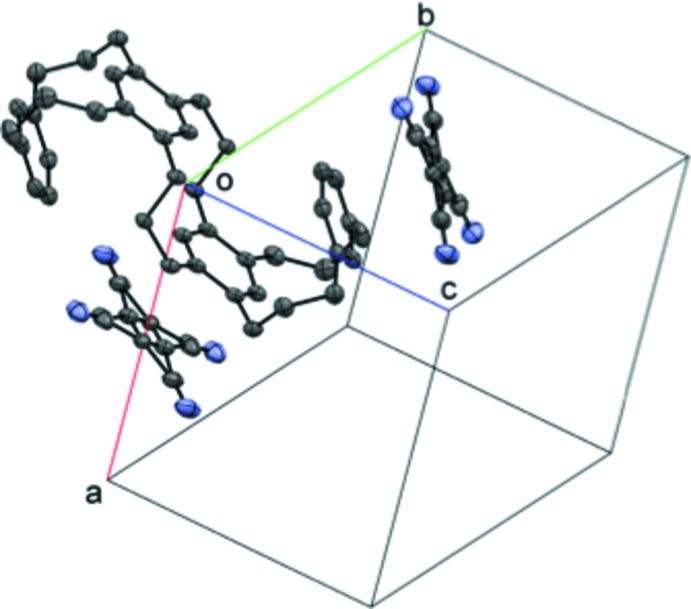
A packing diagram of C42H48.2C6N4.

## supplementary crystallographic information

### 1. Comment

The title complex consists of four-layered [3.3]metacyclophane (MCP) with two tetracyanoethylene molecules (TCNE). The TCNE molecules are stacked on either side of the outer [3.3]MCP units at a distance of 3.19 Å on one side and 3.24 Å on the other, and showed 0.80:0.20 and 0.44:0.56 disorder, respectively.

### 2. Experimental

The complex of single-crystal C_42_H_48_.2C_6_N_4_, consisting of four-layered [3.3]metacyclophane (MCP) with two tetracyanoethylene (TCNE) molecules, was synthesized. The black block crystals were obtained by slow diffusion from 2 m*M* of four-layered [3.3]MCP with 20 m*M* of TCNE in chloroform solution.

### 3. Refinement

All the hydrogen atoms of the compound are fixed geometrically (C—H = 0.93–0.97 Å) and allowed to ride on their parent atoms. Structure was refined with unique reflections and with a cut-off sigma = 2.00.

### Crystal data

**Table:**

C_42_H_48_·2C_6_N_4_	*Z* = 1
*M**_r_* = 809.03	*F*(000) = 428.00
Triclinic, *P*1	*D*_x_ = 1.284 Mg m^−^^3^
Hall symbol: -P 1	Mo *K*α radiation, λ = 0.71075 Å
*a* = 9.563 (3) Å	Cell parameters from 13845 reflections
*b* = 10.101 (4) Å	θ = 3.0–27.5°
*c* = 11.679 (4) Å	µ = 0.08 mm^−^^1^
α = 96.365 (14)°	*T* = 123 K
β = 99.134 (13)°	Block, black
γ = 107.683 (13)°	0.35 × 0.16 × 0.09 mm
*V* = 1045.9 (6) Å^3^	

### Data collection

**Table:**

Rigaku R-AXIS RAPID diffractometer	*R*_int_ = 0.050
ω/2θ scans	θ_max_ = 27.5°
17470 measured reflections	*h* = −12→11
4775 independent reflections	*k* = −13→13
4141 reflections with *F*^2^ > 2σ(*F*^2^)	*l* = −15→15

### Refinement

**Table:**

Refinement on *F*^2^	Secondary atom site location: difference Fourier map
*R*[*F*^2^ > 2σ(*F*^2^)] = 0.060	Hydrogen site location: inferred from neighbouring sites
*wR*(*F*^2^) = 0.178	Only H-atom coordinates refined
*S* = 1.00	*w* = 1/[σ^2^(*F*_o_^2^) + (0.1296*P*)^2^ + 0.1947*P*] where *P* = (*F*_o_^2^ + 2*F*_c_^2^)/3
4775 reflections	(Δ/σ)_max_ < 0.001
394 parameters	Δρ_max_ = 0.80 e Å^−^^3^
0 restraints	Δρ_min_ = −0.50 e Å^−^^3^
Primary atom site location: structure-invariant direct methods	

### Special details

**Table:**

Geometry. ENTER SPECIAL DETAILS OF THE MOLECULAR GEOMETRY
Refinement. Refinement was performed using all reflections. The weighted *R*-factor (*wR*) and goodness of fit (*S*) are based on *F*^2^. *R*-factor (gt) are based on *F*. The threshold expression of *F*^2^ > 2.0 σ(*F*^2^) is used only for calculating *R*-factor (gt).

### Fractional atomic coordinates and isotropic or equivalent isotropic displacement parameters (Å^2^)

**Table:**

	*x*	*y*	*z*	*U*_iso_*/*U*_eq_	Occ. (<1)
N1	0.30526 (16)	0.71021 (14)	0.53324 (14)	0.0447 (4)	
N2	−0.13890 (17)	0.62272 (16)	0.31010 (12)	0.0437 (4)	
N3	0.70465 (15)	0.29951 (14)	0.04664 (13)	0.0426 (4)	
N4	0.63339 (18)	−0.04481 (19)	0.23634 (12)	0.0507 (4)	
C1	0.27496 (16)	0.35932 (14)	0.38821 (12)	0.0292 (3)	
C2	0.17966 (15)	0.23727 (14)	0.41376 (11)	0.0271 (3)	
C3	0.02899 (16)	0.18988 (15)	0.35613 (12)	0.0308 (3)	
C4	−0.02123 (17)	0.26252 (16)	0.27325 (13)	0.0344 (4)	
C5	0.07747 (18)	0.38055 (16)	0.24516 (12)	0.0344 (4)	
C6	0.22797 (17)	0.42945 (14)	0.30050 (13)	0.0314 (4)	
C7	0.3425 (3)	0.54751 (16)	0.26353 (17)	0.0421 (4)	
C8	0.46096 (18)	0.50271 (16)	0.21084 (14)	0.0379 (4)	
C9	0.40369 (16)	0.39146 (14)	0.10058 (13)	0.0306 (3)	
C10	0.33220 (14)	0.23804 (13)	0.11189 (12)	0.0247 (3)	
C11	0.22263 (14)	0.14374 (13)	0.01981 (11)	0.0232 (3)	
C12	0.16183 (14)	0.00361 (13)	0.03416 (11)	0.0237 (3)	
C13	0.19319 (14)	−0.04508 (13)	0.13956 (11)	0.0232 (3)	
C14	0.30253 (14)	0.04905 (13)	0.23209 (11)	0.0235 (3)	
C15	0.37473 (14)	0.18541 (13)	0.21330 (12)	0.0251 (3)	
C16	0.34394 (15)	0.00796 (14)	0.35098 (12)	0.0271 (3)	
C17	0.23470 (17)	0.00835 (14)	0.43550 (12)	0.0309 (3)	
C18	0.23976 (18)	0.15347 (15)	0.49632 (12)	0.0332 (4)	
C19	0.15916 (14)	0.18891 (14)	−0.09117 (11)	0.0248 (3)	
C20	0.11033 (14)	−0.19487 (13)	0.15283 (12)	0.0253 (3)	
C21	−0.04379 (14)	−0.26541 (13)	0.07162 (11)	0.0240 (3)	
C22	0.0677 (9)	0.5125 (7)	0.5331 (7)	0.0274 (14)	0.2000
C23	0.0276 (2)	0.55598 (17)	0.47359 (15)	0.0251 (4)	0.8000
C24	0.18542 (17)	0.63929 (15)	0.51109 (13)	0.0343 (4)	
C25	−0.07150 (17)	0.58885 (15)	0.38230 (12)	0.0332 (4)	
C26	0.5541 (3)	0.0410 (3)	0.0464 (3)	0.0283 (5)	0.5600
C27	0.4895 (4)	−0.0541 (4)	0.0308 (3)	0.0263 (6)	0.4400
C28	0.62892 (17)	0.18694 (17)	0.03210 (17)	0.0426 (4)	
C29	0.58456 (17)	−0.02573 (17)	0.14916 (13)	0.0354 (4)	
H1	0.383 (2)	0.3942 (18)	0.4314 (15)	0.033 (5)*	
H2	−0.045 (3)	0.101 (3)	0.3716 (17)	0.049 (6)*	
H3	−0.131 (3)	0.228 (2)	0.2321 (16)	0.041 (5)*	
H4	0.041 (3)	0.428 (3)	0.1855 (19)	0.050 (6)*	
H5	0.297 (3)	0.587 (3)	0.205 (3)	0.081 (8)*	
H6	0.390 (3)	0.626 (3)	0.327 (2)	0.058 (6)*	
H7	0.534 (3)	0.588 (3)	0.1889 (17)	0.051 (6)*	
H8	0.530 (3)	0.469 (2)	0.2774 (17)	0.042 (5)*	
H9	0.322 (3)	0.419 (3)	0.0470 (18)	0.051 (6)*	
H10	0.488 (2)	0.3976 (19)	0.0554 (16)	0.043 (5)*	
H11	0.0882 (17)	−0.0620 (16)	−0.0336 (14)	0.021 (4)*	
H12	0.4578 (18)	0.2534 (17)	0.2809 (14)	0.027 (4)*	
H13	0.452 (2)	0.0727 (18)	0.3944 (15)	0.033 (5)*	
H14	0.3556 (17)	−0.0871 (17)	0.3366 (13)	0.024 (4)*	
H15	0.260 (2)	−0.0488 (19)	0.4963 (16)	0.038 (5)*	
H16	0.125 (2)	−0.0487 (19)	0.3960 (16)	0.037 (5)*	
H17	0.179 (3)	0.138 (2)	0.5597 (18)	0.048 (5)*	
H18	0.352 (3)	0.212 (3)	0.5384 (18)	0.050 (6)*	
H19	0.1086 (18)	0.0987 (18)	−0.1549 (14)	0.029 (4)*	
H20	0.242 (2)	0.2516 (18)	−0.1231 (15)	0.034 (5)*	
H21	0.0979 (19)	−0.1933 (17)	0.2372 (14)	0.029 (4)*	
H22	0.1777 (19)	−0.2508 (18)	0.1392 (15)	0.035 (5)*	
H23	−0.0359 (18)	−0.2743 (17)	−0.0123 (15)	0.029 (4)*	
H24	−0.0813 (19)	−0.3631 (18)	0.0886 (14)	0.028 (4)*	

### Atomic displacement parameters (Å^2^)

**Table:**

	*U*^11^	*U*^22^	*U*^33^	*U*^12^	*U*^13^	*U*^23^
N1	0.0341 (8)	0.0348 (7)	0.0587 (9)	0.0038 (6)	0.0098 (6)	0.0017 (6)
N2	0.0523 (9)	0.0596 (9)	0.0346 (7)	0.0334 (7)	0.0143 (6)	0.0209 (7)
N3	0.0335 (7)	0.0360 (7)	0.0599 (9)	0.0112 (6)	0.0152 (6)	0.0066 (6)
N4	0.0539 (9)	0.0834 (12)	0.0354 (8)	0.0470 (9)	0.0129 (7)	0.0190 (7)
C1	0.0316 (8)	0.0267 (7)	0.0322 (7)	0.0119 (6)	0.0093 (6)	0.0056 (6)
C2	0.0324 (7)	0.0262 (7)	0.0252 (7)	0.0116 (6)	0.0086 (5)	0.0048 (5)
C3	0.0321 (8)	0.0283 (7)	0.0326 (7)	0.0101 (6)	0.0100 (6)	0.0023 (6)
C4	0.0354 (8)	0.0381 (8)	0.0310 (7)	0.0181 (6)	0.0033 (6)	−0.0021 (6)
C5	0.0500 (9)	0.0357 (8)	0.0272 (7)	0.0266 (7)	0.0097 (6)	0.0064 (6)
C6	0.0422 (8)	0.0266 (7)	0.0336 (7)	0.0178 (6)	0.0160 (6)	0.0077 (6)
C7	0.0601 (11)	0.0261 (7)	0.0492 (10)	0.0170 (7)	0.0262 (8)	0.0138 (7)
C8	0.0360 (8)	0.0302 (7)	0.0450 (9)	0.0051 (6)	0.0083 (7)	0.0134 (7)
C9	0.0333 (8)	0.0256 (7)	0.0385 (8)	0.0108 (6)	0.0145 (6)	0.0144 (6)
C10	0.0235 (7)	0.0250 (6)	0.0318 (7)	0.0118 (5)	0.0111 (5)	0.0119 (5)
C11	0.0222 (6)	0.0262 (6)	0.0288 (7)	0.0136 (5)	0.0102 (5)	0.0121 (5)
C12	0.0211 (6)	0.0263 (7)	0.0285 (7)	0.0113 (5)	0.0080 (5)	0.0104 (5)
C13	0.0214 (6)	0.0251 (6)	0.0294 (7)	0.0124 (5)	0.0087 (5)	0.0111 (5)
C14	0.0219 (6)	0.0265 (6)	0.0279 (7)	0.0130 (5)	0.0081 (5)	0.0104 (5)
C15	0.0235 (7)	0.0260 (7)	0.0296 (7)	0.0110 (5)	0.0079 (5)	0.0088 (5)
C16	0.0282 (7)	0.0271 (7)	0.0285 (7)	0.0115 (5)	0.0043 (5)	0.0104 (5)
C17	0.0395 (8)	0.0282 (7)	0.0295 (7)	0.0137 (6)	0.0098 (6)	0.0119 (6)
C18	0.0447 (9)	0.0305 (7)	0.0274 (7)	0.0153 (6)	0.0073 (6)	0.0088 (6)
C19	0.0249 (7)	0.0279 (7)	0.0275 (7)	0.0125 (5)	0.0087 (5)	0.0126 (5)
C20	0.0249 (7)	0.0249 (6)	0.0308 (7)	0.0115 (5)	0.0073 (5)	0.0126 (5)
C21	0.0266 (7)	0.0223 (6)	0.0268 (7)	0.0113 (5)	0.0065 (5)	0.0086 (5)
C22	0.040 (5)	0.017 (3)	0.031 (4)	0.010 (4)	0.019 (3)	0.012 (3)
C23	0.0266 (10)	0.0231 (8)	0.0266 (9)	0.0084 (8)	0.0064 (7)	0.0061 (7)
C24	0.0343 (8)	0.0280 (7)	0.0386 (8)	0.0063 (6)	0.0121 (6)	0.0022 (6)
C25	0.0437 (9)	0.0334 (7)	0.0293 (7)	0.0195 (6)	0.0101 (6)	0.0101 (6)
C26	0.0248 (12)	0.0312 (16)	0.0336 (16)	0.0135 (12)	0.0075 (11)	0.0101 (11)
C27	0.0206 (14)	0.0318 (19)	0.0283 (17)	0.0114 (14)	0.0046 (12)	0.0046 (14)
C28	0.0300 (8)	0.0343 (8)	0.0663 (11)	0.0112 (7)	0.0186 (7)	0.0048 (8)
C29	0.0316 (8)	0.0508 (9)	0.0325 (8)	0.0241 (7)	0.0081 (6)	0.0110 (7)

### Geometric parameters (Å, º)

**Table:**

N1—C24	1.1223 (19)	C23—C25	1.447 (3)
N2—C25	1.126 (3)	C26—C26^iii^	1.362 (4)
N3—C28	1.124 (2)	C26—C27^iii^	0.972 (5)
N4—C29	1.113 (3)	C26—C28	1.472 (4)
C1—C2	1.3873 (19)	C26—C29	1.472 (4)
C1—C6	1.400 (3)	C27—C27^iii^	1.356 (6)
C2—C3	1.4000 (19)	C27—C28^iii^	1.488 (4)
C2—C18	1.509 (3)	C27—C29	1.475 (4)
C3—C4	1.389 (3)	C1—H1	1.007 (17)
C4—C5	1.386 (3)	C3—H2	1.01 (2)
C5—C6	1.391 (3)	C4—H3	1.015 (19)
C6—C7	1.510 (3)	C5—H4	0.97 (3)
C7—C8	1.529 (3)	C7—H5	0.95 (3)
C8—C9	1.517 (2)	C7—H6	0.96 (2)
C9—C10	1.5221 (19)	C8—H7	1.02 (2)
C10—C11	1.4037 (16)	C8—H8	1.09 (2)
C10—C15	1.403 (2)	C9—H9	1.05 (3)
C11—C12	1.3975 (19)	C9—H10	1.02 (2)
C11—C19	1.519 (2)	C12—H11	0.992 (13)
C12—C13	1.398 (2)	C15—H12	1.037 (14)
C13—C14	1.4052 (16)	C16—H13	1.047 (15)
C13—C20	1.5178 (18)	C16—H14	0.999 (18)
C14—C15	1.3997 (18)	C17—H15	1.01 (2)
C14—C16	1.520 (2)	C17—H16	1.029 (17)
C16—C17	1.547 (3)	C18—H17	1.01 (3)
C17—C18	1.540 (3)	C18—H18	1.060 (19)
C19—C21^i^	1.557 (3)	C19—H19	1.042 (15)
C20—C21	1.5323 (17)	C19—H20	1.006 (18)
C22—C22^ii^	1.333 (11)	C20—H21	1.010 (18)
C22—C24	1.504 (7)	C20—H22	1.00 (2)
C22—C25^ii^	1.504 (8)	C21—H23	0.991 (18)
C23—C23^ii^	1.354 (3)	C21—H24	0.996 (18)
C23—C24	1.454 (3)		
			
N1···C22^ii^	3.499 (8)	C20···H14	2.762 (14)
N1···C23^ii^	3.470 (3)	C20···H16	3.013 (18)
N1···C25	3.523 (3)	C20···H19^i^	2.56 (2)
N1···C25^ii^	3.515 (3)	C20···H20^i^	3.198 (19)
N2···C22	3.503 (8)	C21···H4^i^	3.27 (3)
N2···C23^ii^	3.478 (3)	C21···H9^i^	2.697 (19)
N2···C24	3.528 (3)	C21···H11	2.608 (16)
N2···C24^ii^	3.517 (3)	C21···H11^i^	3.526 (18)
N3···C26^iii^	3.504 (3)	H1···H5	3.59 (4)
N3···C27	3.506 (4)	H1···H6	2.74 (4)
N3···C29	3.544 (3)	H1···H8	2.50 (3)
N3···C29^iii^	3.531 (2)	H1···H12	2.47 (3)
N4···C26^iii^	3.509 (4)	H1···H13	3.50 (3)
N4···C27^iii^	3.514 (4)	H1···H17	3.39 (3)
N4···C28	3.527 (3)	H1···H18	2.31 (3)
N4···C28^iii^	3.535 (3)	H2···H3	2.38 (3)
C1···C4	2.757 (2)	H2···H16	2.54 (4)
C1···C8	3.134 (3)	H2···H17	2.72 (3)
C1···C10	3.508 (3)	H2···H19^i^	2.91 (3)
C1···C14	3.550 (3)	H3···H4	2.36 (3)
C1···C15	3.002 (3)	H3···H11^i^	2.86 (3)
C1···C17	3.563 (3)	H3···H19^i^	3.40 (3)
C2···C5	2.794 (3)	H3···H23^i^	3.23 (3)
C2···C14	3.279 (3)	H4···H5	2.44 (3)
C2···C15	3.308 (3)	H4···H6	3.37 (3)
C2···C16	3.251 (3)	H4···H9	3.37 (4)
C3···C6	2.800 (2)	H4···H23^i^	2.39 (3)
C3···C17	3.175 (3)	H4···H24^i^	3.30 (3)
C5···C8	3.600 (3)	H5···H7	2.30 (4)
C6···C9	3.140 (3)	H5···H8	2.89 (4)
C6···C10	3.241 (3)	H5···H9	2.45 (4)
C6···C15	3.329 (3)	H5···H10	3.53 (4)
C7···C10	3.383 (3)	H6···H7	2.35 (4)
C8···C15	3.062 (3)	H6···H8	2.44 (4)
C9···C19	3.0140 (18)	H6···H9	3.53 (3)
C9···C21^i^	3.476 (2)	H7···H9	2.44 (3)
C10···C13	2.843 (2)	H7···H10	2.22 (3)
C10···C21^i^	3.316 (2)	H7···H12	3.56 (3)
C11···C12^i^	3.444 (2)	H8···H9	2.97 (3)
C11···C14	2.831 (2)	H8···H10	2.55 (3)
C12···C12^i^	3.048 (2)	H8···H12	2.09 (3)
C12···C15	2.7237 (17)	H9···H20	2.32 (3)
C12···C19^i^	3.310 (2)	H9···H23^i^	2.63 (3)
C12···C21	2.9550 (19)	H9···H24^i^	2.45 (3)
C12···C21^i^	3.448 (3)	H10···H12	3.16 (3)
C13···C17	3.379 (3)	H10···H20	2.78 (3)
C13···C19^i^	3.1660 (19)	H11···H11^i^	2.55 (3)
C14···C18	3.354 (3)	H11···H19	2.26 (3)
C15···C17	3.522 (3)	H11···H19^i^	3.10 (3)
C16···C20	3.0117 (18)	H11···H20	3.42 (3)
C17···C20	3.506 (2)	H11···H21	3.56 (3)
N1···C6	3.515 (3)	H11···H22	3.12 (3)
N1···C7	3.509 (3)	H11···H23	2.18 (3)
N1···C17^iv^	3.547 (3)	H11···H23^i^	3.58 (3)
N2···C2^ii^	3.504 (3)	H11···H24	3.55 (3)
N2···C8^v^	3.594 (3)	H12···H13	2.36 (3)
N2···C18^ii^	3.469 (3)	H12···H14	3.44 (3)
N2···C19^vi^	3.368 (3)	H12···H18	3.35 (3)
N2···C20^iv^	3.453 (3)	H13···H15	2.43 (3)
N2···C21^iv^	3.277 (3)	H13···H16	2.99 (3)
N3···C4^vii^	3.553 (3)	H13···H18	2.55 (3)
N3···C9	3.408 (3)	H14···H15	2.25 (3)
N3···C20^iii^	3.402 (3)	H14···H16	2.53 (3)
N4···C14	3.563 (3)	H14···H21	2.40 (2)
N4···C16	3.414 (3)	H14···H22	2.67 (2)
N4···C18^viii^	3.576 (3)	H15···H17	2.33 (4)
N4···C19^iii^	3.347 (3)	H15···H18	2.48 (3)
C1···C22	3.396 (9)	H15···H21	3.14 (3)
C1···C24	3.428 (3)	H16···H17	2.40 (3)
C2···N2^ii^	3.504 (3)	H16···H18	2.97 (3)
C2···C22	3.499 (9)	H16···H19^i^	3.19 (3)
C2···C23^ii^	3.571 (3)	H16···H21	2.16 (3)
C2···C25^ii^	3.284 (3)	H19···H21^i^	2.55 (3)
C3···C22	3.544 (8)	H19···H22^i^	3.54 (3)
C3···C22^ii^	3.573 (9)	H19···H23^i^	2.80 (3)
C3···C23^ii^	3.300 (3)	H19···H24^i^	2.80 (3)
C3···C24^ii^	3.486 (3)	H20···H21^i^	3.16 (3)
C3···C25^ii^	3.469 (3)	H20···H23^i^	2.76 (3)
C4···N3^v^	3.553 (3)	H20···H24^i^	2.22 (3)
C4···C22	3.534 (7)	H21···H23	2.91 (3)
C4···C22^ii^	3.204 (8)	H21···H24	2.346 (19)
C4···C23	3.428 (3)	H22···H23	2.41 (3)
C4···C23^ii^	3.321 (3)	H22···H24	2.33 (3)
C4···C24^ii^	3.382 (3)	N1···H1^x^	3.44 (2)
C4···C25	3.598 (3)	N1···H3^ii^	3.52 (3)
C5···C22	3.504 (8)	N1···H6	2.79 (3)
C5···C22^ii^	3.351 (9)	N1···H8^x^	3.49 (3)
C5···C23	3.218 (3)	N1···H12^x^	2.781 (16)
C5···C25	3.289 (3)	N1···H13^x^	2.607 (15)
C6···N1	3.515 (3)	N1···H14^iv^	3.239 (17)
C6···C22	3.468 (9)	N1···H15^iv^	2.67 (2)
C6···C23	3.397 (3)	N1···H18^x^	3.39 (3)
C6···C24	3.224 (3)	N2···H4	3.34 (3)
C7···N1	3.509 (3)	N2···H7^v^	3.12 (2)
C8···N2^vii^	3.594 (3)	N2···H8^v^	3.010 (19)
C9···N3	3.408 (3)	N2···H16^iv^	3.431 (16)
C9···C28	3.526 (3)	N2···H17^ii^	2.87 (3)
C10···C26	3.434 (4)	N2···H18^ii^	3.55 (3)
C10···C27^iii^	3.358 (5)	N2···H19^vi^	3.477 (18)
C10···C28	3.288 (3)	N2···H20^vi^	2.838 (19)
C11···C26^iii^	3.356 (4)	N2···H21^iv^	2.777 (17)
C11···C27^iii^	3.265 (5)	N2···H24^iv^	2.740 (17)
C11···C29^iii^	3.269 (3)	N3···H3^vii^	2.76 (2)
C12···C26^iii^	3.151 (4)	N3···H4^vii^	3.16 (2)
C12···C27	3.362 (5)	N3···H5^xi^	3.27 (3)
C12···C27^iii^	3.436 (4)	N3···H9^xi^	3.22 (3)
C12···C28^iii^	3.288 (3)	N3···H10	2.56 (3)
C12···C29^iii^	3.456 (3)	N3···H11^iii^	3.550 (19)
C13···C26^iii^	3.491 (4)	N3···H22^iii^	2.663 (19)
C13···C27	3.305 (4)	N3···H23^iii^	3.336 (19)
C13···C28^iii^	3.300 (3)	N3···H24^xii^	3.334 (16)
C14···N4	3.563 (3)	N4···H2^vii^	3.055 (19)
C14···C26	3.497 (4)	N4···H3^vii^	2.996 (18)
C14···C27	3.426 (5)	N4···H7^ix^	3.49 (2)
C14···C29	3.269 (3)	N4···H13	3.10 (2)
C15···C26	3.289 (4)	N4···H14	3.003 (17)
C15···C27	3.580 (5)	N4···H15^viii^	3.071 (17)
C15···C27^iii^	3.599 (4)	N4···H17^viii^	3.15 (3)
C15···C28	3.465 (3)	N4···H18^viii^	3.29 (3)
C15···C29	3.444 (3)	N4···H19^iii^	2.955 (19)
C16···N4	3.414 (3)	N4···H20^iii^	2.99 (2)
C17···N1^ix^	3.547 (3)	C3···H15^xiii^	3.508 (19)
C18···N2^ii^	3.469 (3)	C7···H18^x^	3.476 (17)
C18···N4^viii^	3.576 (3)	C7···H22^iv^	3.26 (2)
C19···N2^vi^	3.368 (3)	C8···H10^xi^	3.435 (19)
C19···N4^iii^	3.347 (3)	C8···H20^xi^	3.532 (18)
C19···C29^iii^	3.452 (3)	C9···H7^xi^	3.54 (2)
C20···N2^ix^	3.453 (3)	C9···H9^xi^	3.55 (3)
C20···N3^iii^	3.402 (3)	C9···H10^xi^	3.008 (19)
C20···C28^iii^	3.538 (3)	C17···H2^xiii^	3.20 (3)
C21···N2^ix^	3.277 (3)	C18···H2^xiii^	3.37 (2)
C22···C1	3.396 (9)	C19···H7^xi^	3.55 (2)
C22···C2	3.499 (9)	C20···H5^ix^	3.28 (4)
C22···C3	3.544 (8)	C22···H3^ii^	3.42 (2)
C22···C3^ii^	3.573 (9)	C23···H4	3.50 (3)
C22···C4	3.534 (7)	C24···H2^ii^	3.53 (3)
C22···C4^ii^	3.204 (8)	C24···H3^ii^	3.33 (2)
C22···C5	3.504 (8)	C24···H6	3.15 (3)
C22···C5^ii^	3.351 (9)	C24···H15^iv^	3.043 (19)
C22···C6	3.468 (9)	C25···H4	3.18 (3)
C23···C2^ii^	3.571 (3)	C25···H8^v^	3.586 (19)
C23···C3^ii^	3.300 (3)	C25···H16^iv^	3.544 (18)
C23···C4	3.428 (3)	C25···H17^ii^	3.27 (3)
C23···C4^ii^	3.321 (3)	C25···H21^iv^	3.142 (17)
C23···C5	3.218 (3)	C25···H24^iv^	3.508 (17)
C23···C6	3.397 (3)	C26···H3^vii^	3.304 (17)
C24···C1	3.428 (3)	C26···H11^iii^	3.391 (18)
C24···C3^ii^	3.486 (3)	C27···H10^iii^	3.58 (2)
C24···C4^ii^	3.382 (3)	C27···H22	3.549 (18)
C24···C6	3.224 (3)	C28···H3^vii^	2.900 (19)
C25···C2^ii^	3.284 (3)	C28···H10	2.85 (3)
C25···C3^ii^	3.469 (3)	C28···H11^iii^	3.313 (19)
C25···C4	3.598 (3)	C28···H22^iii^	2.927 (19)
C25···C5	3.289 (3)	C29···H3^vii^	3.049 (17)
C26···C10	3.434 (4)	C29···H11^iii^	3.510 (17)
C26···C11^iii^	3.356 (4)	C29···H13	3.479 (19)
C26···C12^iii^	3.151 (4)	C29···H14	3.312 (17)
C26···C13^iii^	3.491 (4)	C29···H19^iii^	3.228 (19)
C26···C14	3.497 (4)	C29···H20^iii^	3.22 (3)
C26···C15	3.289 (4)	H1···N1^x^	3.44 (2)
C27···C10^iii^	3.358 (5)	H1···H1^x^	2.71 (2)
C27···C11^iii^	3.265 (5)	H1···H6^x^	3.34 (3)
C27···C12	3.362 (5)	H1···H8^x^	3.39 (3)
C27···C12^iii^	3.436 (4)	H2···N4^v^	3.055 (19)
C27···C13	3.305 (4)	H2···C17^xiii^	3.20 (3)
C27···C14	3.426 (5)	H2···C18^xiii^	3.37 (2)
C27···C15	3.580 (5)	H2···C24^ii^	3.53 (3)
C27···C15^iii^	3.599 (4)	H2···H15^xiii^	2.73 (3)
C28···C9	3.526 (3)	H2···H16^xiii^	3.00 (3)
C28···C10	3.288 (3)	H2···H17^xiii^	2.65 (3)
C28···C12^iii^	3.288 (3)	H3···N1^ii^	3.52 (3)
C28···C13^iii^	3.300 (3)	H3···N3^v^	2.76 (2)
C28···C15	3.465 (3)	H3···N4^v^	2.996 (18)
C28···C20^iii^	3.538 (3)	H3···C22^ii^	3.42 (2)
C29···C11^iii^	3.269 (3)	H3···C24^ii^	3.33 (2)
C29···C12^iii^	3.456 (3)	H3···C26^v^	3.304 (17)
C29···C14	3.269 (3)	H3···C28^v^	2.900 (19)
C29···C15	3.444 (3)	H3···C29^v^	3.049 (17)
C29···C19^iii^	3.452 (3)	H4···N2	3.34 (3)
C1···H2	3.321 (19)	H4···N3^v^	3.16 (2)
C1···H4	3.27 (3)	H4···C23	3.50 (3)
C1···H5	3.30 (3)	H4···C25	3.18 (3)
C1···H6	2.80 (3)	H4···H22^iv^	3.25 (3)
C1···H8	2.92 (2)	H4···H24^iv^	2.95 (4)
C1···H12	2.70 (2)	H5···N3^xi^	3.27 (3)
C1···H17	3.19 (3)	H5···C20^iv^	3.28 (4)
C1···H18	2.58 (3)	H5···H14^iv^	3.32 (4)
C2···H3	3.33 (2)	H5···H21^iv^	3.36 (4)
C2···H12	3.257 (19)	H5···H22^iv^	2.39 (4)
C2···H13	3.51 (2)	H6···N1	2.79 (3)
C2···H15	3.41 (2)	H6···C24	3.15 (3)
C2···H16	2.75 (2)	H6···H1^x^	3.34 (3)
C3···H1	3.299 (16)	H6···H14^iv^	3.01 (4)
C3···H4	3.28 (3)	H6···H18^x^	2.64 (3)
C3···H16	2.89 (3)	H6···H22^iv^	3.35 (4)
C3···H17	2.77 (2)	H7···N2^vii^	3.12 (2)
C3···H18	3.39 (2)	H7···N4^iv^	3.49 (2)
C3···H19^i^	3.304 (15)	H7···C9^xi^	3.54 (2)
C4···H11^i^	3.119 (15)	H7···C19^xi^	3.55 (2)
C4···H19^i^	3.547 (17)	H7···H9^xi^	3.27 (4)
C4···H23^i^	3.189 (18)	H7···H10^xi^	2.85 (3)
C5···H1	3.308 (18)	H7···H18^x^	3.40 (3)
C5···H2	3.31 (2)	H7···H20^xi^	2.55 (3)
C5···H5	2.62 (3)	H8···N1^x^	3.49 (3)
C5···H6	3.17 (2)	H8···N2^vii^	3.010 (19)
C5···H9	3.52 (3)	H8···C25^vii^	3.586 (19)
C5···H11^i^	3.587 (14)	H8···H1^x^	3.39 (3)
C5···H23^i^	2.731 (17)	H8···H18^x^	3.42 (3)
C6···H3	3.327 (18)	H9···N3^xi^	3.22 (3)
C6···H7	3.41 (2)	H9···C9^xi^	3.55 (3)
C6···H8	2.86 (2)	H9···H7^xi^	3.27 (4)
C6···H9	3.23 (3)	H9···H10^xi^	2.70 (3)
C6···H12	3.24 (2)	H10···N3	2.56 (3)
C6···H23^i^	3.504 (15)	H10···C8^xi^	3.435 (19)
C7···H1	2.681 (19)	H10···C9^xi^	3.008 (19)
C7···H4	2.72 (2)	H10···C27^iii^	3.58 (2)
C7···H9	2.66 (2)	H10···C28	2.85 (3)
C7···H10	3.42 (2)	H10···H7^xi^	2.85 (3)
C7···H12	3.484 (19)	H10···H9^xi^	2.70 (3)
C8···H1	3.023 (19)	H10···H10^xi^	2.53 (3)
C8···H12	2.726 (18)	H11···N3^iii^	3.550 (19)
C9···H5	2.74 (3)	H11···C26^iii^	3.391 (18)
C9···H6	3.39 (3)	H11···C28^iii^	3.313 (19)
C9···H12	2.719 (18)	H11···C29^iii^	3.510 (17)
C9···H20	2.789 (15)	H12···N1^x^	2.781 (16)
C9···H23^i^	3.299 (16)	H13···N1^x^	2.607 (15)
C9···H24^i^	3.410 (17)	H13···N4	3.10 (2)
C10···H7	3.411 (19)	H13···C29	3.479 (19)
C10···H8	2.817 (16)	H13···H13^viii^	3.16 (3)
C10···H11	3.289 (13)	H13···H14^viii^	3.34 (3)
C10···H19	3.368 (15)	H13···H15^viii^	2.94 (3)
C10···H20	2.773 (18)	H14···N1^ix^	3.239 (17)
C10···H23^i^	3.033 (18)	H14···N4	3.003 (17)
C11···H9	2.62 (2)	H14···C29	3.312 (17)
C11···H10	2.944 (17)	H14···H5^ix^	3.32 (4)
C11···H11^i^	2.870 (16)	H14···H6^ix^	3.01 (4)
C11···H12	3.338 (15)	H14···H13^viii^	3.34 (3)
C11···H23^i^	2.52 (2)	H14···H18^viii^	3.58 (3)
C11···H24^i^	3.20 (2)	H15···N1^ix^	2.67 (2)
C12···H3^i^	3.592 (19)	H15···N4^viii^	3.071 (17)
C12···H11^i^	2.630 (18)	H15···C3^xiii^	3.508 (19)
C12···H19	2.543 (18)	H15···C24^ix^	3.043 (19)
C12···H19^i^	3.099 (18)	H15···H2^xiii^	2.73 (3)
C12···H20	3.245 (19)	H15···H13^viii^	2.94 (3)
C12···H21	3.274 (18)	H16···N2^ix^	3.431 (16)
C12···H22	3.00 (2)	H16···C25^ix^	3.544 (18)
C12···H23	2.804 (15)	H16···H2^xiii^	3.00 (3)
C12···H23^i^	3.324 (19)	H16···H17^xiii^	2.93 (3)
C13···H11^i^	3.309 (18)	H17···N2^ii^	2.87 (3)
C13···H12	3.339 (14)	H17···N4^viii^	3.15 (3)
C13···H13	3.388 (16)	H17···C25^ii^	3.27 (3)
C13···H14	2.715 (16)	H17···H2^xiii^	2.65 (3)
C13···H16	3.165 (19)	H17···H16^xiii^	2.93 (3)
C13···H19^i^	2.809 (18)	H17···H19^xiv^	3.54 (3)
C13···H23	2.842 (14)	H18···N1^x^	3.39 (3)
C13···H24	3.394 (15)	H18···N2^ii^	3.55 (3)
C14···H11	3.302 (14)	H18···N4^viii^	3.29 (3)
C14···H15	3.390 (19)	H18···C7^x^	3.476 (17)
C14···H16	2.82 (2)	H18···H6^x^	2.64 (3)
C14···H21	2.643 (15)	H18···H7^x^	3.40 (3)
C14···H22	2.905 (17)	H18···H8^x^	3.42 (3)
C15···H1	3.098 (18)	H18···H14^viii^	3.58 (3)
C15···H8	2.754 (18)	H19···N2^vi^	3.477 (18)
C15···H9	3.32 (3)	H19···N4^iii^	2.955 (19)
C15···H10	3.044 (19)	H19···C29^iii^	3.228 (19)
C15···H13	2.620 (19)	H19···H17^xv^	3.54 (3)
C15···H14	3.215 (17)	H20···N2^vi^	2.838 (19)
C16···H12	2.663 (17)	H20···N4^iii^	2.99 (2)
C16···H17	3.45 (3)	H20···C8^xi^	3.532 (18)
C16···H18	2.81 (2)	H20···C29^iii^	3.22 (3)
C16···H21	2.634 (14)	H20···H7^xi^	2.55 (3)
C16···H22	3.205 (15)	H21···N2^ix^	2.777 (17)
C17···H2	3.10 (3)	H21···C25^ix^	3.142 (17)
C17···H12	3.597 (17)	H21···H5^ix^	3.36 (4)
C17···H21	2.756 (15)	H22···N3^iii^	2.663 (19)
C18···H1	2.659 (18)	H22···C7^ix^	3.26 (2)
C18···H2	2.74 (2)	H22···C27	3.549 (18)
C18···H12	3.556 (18)	H22···C28^iii^	2.927 (19)
C18···H13	2.78 (2)	H22···H4^ix^	3.25 (3)
C18···H14	3.449 (18)	H22···H5^ix^	2.39 (4)
C19···H9	2.574 (18)	H22···H6^ix^	3.35 (4)
C19···H10	3.277 (16)	H23···N3^iii^	3.336 (19)
C19···H11	2.610 (16)	H24···N2^ix^	2.740 (17)
C19···H11^i^	3.005 (17)	H24···N3^xvi^	3.334 (16)
C19···H21^i^	2.776 (17)	H24···C25^ix^	3.508 (17)
C19···H22^i^	3.44 (2)	H24···H4^ix^	2.95 (4)
C20···H11	2.695 (17)		
			
C2—C1—C6	122.08 (13)	N4—C29—C26	162.91 (18)
C1—C2—C3	118.44 (14)	N4—C29—C27	159.5 (2)
C1—C2—C18	120.43 (13)	C26—C29—C27	37.62 (17)
C3—C2—C18	121.03 (13)	C2—C1—H1	118.1 (11)
C2—C3—C4	120.06 (13)	C6—C1—H1	119.8 (11)
C3—C4—C5	120.49 (14)	C2—C3—H2	121.3 (13)
C4—C5—C6	120.66 (15)	C4—C3—H2	118.6 (12)
C1—C6—C5	118.04 (13)	C3—C4—H3	119.8 (12)
C1—C6—C7	119.43 (14)	C5—C4—H3	119.7 (12)
C5—C6—C7	122.39 (15)	C4—C5—H4	119.3 (12)
C6—C7—C8	114.34 (15)	C6—C5—H4	120.1 (12)
C7—C8—C9	116.32 (13)	C6—C7—H5	111.3 (15)
C8—C9—C10	119.05 (14)	C6—C7—H6	111.8 (15)
C9—C10—C11	120.79 (13)	C8—C7—H5	105.0 (19)
C9—C10—C15	121.46 (11)	C8—C7—H6	109.9 (16)
C11—C10—C15	117.74 (12)	H5—C7—H6	104 (3)
C10—C11—C12	118.50 (13)	C7—C8—H7	109.4 (14)
C10—C11—C19	123.07 (12)	C7—C8—H8	110.1 (12)
C12—C11—C19	118.29 (10)	C9—C8—H7	106.1 (11)
C11—C12—C13	123.50 (11)	C9—C8—H8	110.0 (10)
C12—C13—C14	117.90 (12)	H7—C8—H8	104.2 (16)
C12—C13—C20	120.57 (10)	C8—C9—H9	106.3 (12)
C14—C13—C20	121.52 (12)	C8—C9—H10	109.4 (9)
C13—C14—C15	118.34 (13)	C10—C9—H9	106.8 (11)
C13—C14—C16	122.34 (12)	C10—C9—H10	108.3 (11)
C15—C14—C16	119.33 (10)	H9—C9—H10	106.2 (17)
C10—C15—C14	123.34 (11)	C11—C12—H11	117.0 (10)
C14—C16—C17	116.14 (13)	C13—C12—H11	119.5 (10)
C16—C17—C18	116.71 (12)	C10—C15—H12	118.4 (10)
C2—C18—C17	113.70 (12)	C14—C15—H12	118.0 (10)
C11—C19—C21^i^	112.79 (12)	C14—C16—H13	110.5 (10)
C13—C20—C21	116.02 (12)	C14—C16—H14	107.8 (9)
C19^i^—C21—C20	113.53 (11)	C17—C16—H13	108.5 (11)
C22^ii^—C22—C24	113.7 (7)	C17—C16—H14	110.1 (10)
C22^ii^—C22—C25^ii^	113.6 (6)	H13—C16—H14	103.0 (14)
C24—C22—C25^ii^	132.7 (6)	C16—C17—H15	105.0 (12)
C23^ii^—C23—C24	118.44 (17)	C16—C17—H16	112.2 (11)
C23^ii^—C23—C25	119.30 (15)	C18—C17—H15	110.0 (11)
C24—C23—C25	122.26 (15)	C18—C17—H16	108.7 (12)
N1—C24—C22	148.2 (4)	H15—C17—H16	103.3 (15)
N1—C24—C23	174.27 (19)	C2—C18—H17	109.3 (14)
C22—C24—C23	37.5 (4)	C2—C18—H18	109.4 (13)
N2—C25—C22^ii^	148.1 (3)	C17—C18—H17	107.9 (12)
N2—C25—C23	174.49 (16)	C17—C18—H18	109.1 (13)
C22^ii^—C25—C23	37.4 (3)	H17—C18—H18	107.2 (16)
C26^iii^—C26—C27^iii^	44.2 (3)	C11—C19—H19	107.6 (10)
C26^iii^—C26—C28	116.0 (3)	C11—C19—H20	110.4 (11)
C26^iii^—C26—C29	116.9 (3)	C21^i^—C19—H19	110.1 (11)
C27^iii^—C26—C28	71.8 (3)	C21^i^—C19—H20	109.6 (12)
C27^iii^—C26—C29	161.1 (3)	H19—C19—H20	106.1 (14)
C28—C26—C29	127.10 (18)	C13—C20—H21	107.6 (9)
C26^iii^—C27—C27^iii^	44.5 (3)	C13—C20—H22	106.4 (10)
C26^iii^—C27—C28^iii^	69.9 (3)	C21—C20—H21	108.8 (9)
C26^iii^—C27—C29	161.3 (4)	C21—C20—H22	110.0 (9)
C27^iii^—C27—C28^iii^	114.4 (3)	H21—C20—H22	107.8 (15)
C27^iii^—C27—C29	116.8 (3)	C19^i^—C21—H23	109.4 (12)
C28^iii^—C27—C29	128.8 (3)	C19^i^—C21—H24	109.3 (12)
N3—C28—C26	163.7 (2)	C20—C21—H23	111.4 (9)
N3—C28—C27^iii^	157.9 (3)	C20—C21—H24	106.3 (9)
C26—C28—C27^iii^	38.33 (18)	H23—C21—H24	106.6 (13)
			
C2—C1—C6—C5	5.5 (3)	C22^ii^—C22—C24—N1	179.6 (5)
C2—C1—C6—C7	−170.23 (13)	C22^ii^—C22—C24—C23	−0.7 (4)
C6—C1—C2—C3	−5.2 (3)	C24—C22—C22^ii^—C25	1.8 (9)
C6—C1—C2—C18	171.25 (13)	C22^ii^—C22—C25^ii^—N2^ii^	−178.7 (5)
C1—C2—C3—C4	1.7 (2)	C22^ii^—C22—C25^ii^—C23^ii^	0.7 (4)
C1—C2—C18—C17	−111.99 (14)	C25^ii^—C22—C22^ii^—C24^ii^	−1.8 (9)
C3—C2—C18—C17	64.33 (19)	C24—C22—C25^ii^—N2^ii^	−1.0 (13)
C18—C2—C3—C4	−174.72 (13)	C24—C22—C25^ii^—C23^ii^	178.4 (11)
C2—C3—C4—C5	1.3 (3)	C25^ii^—C22—C24—N1	1.9 (13)
C3—C4—C5—C6	−0.9 (3)	C25^ii^—C22—C24—C23	−178.4 (11)
C4—C5—C6—C1	−2.4 (3)	C23^ii^—C23—C24—C22	0.71 (12)
C4—C5—C6—C7	173.19 (14)	C24—C23—C23^ii^—C25^ii^	0.0 (3)
C1—C6—C7—C8	61.87 (19)	C23^ii^—C23—C25—C22^ii^	0.72 (12)
C5—C6—C7—C8	−113.71 (18)	C25—C23—C23^ii^—C24^ii^	−0.0 (3)
C6—C7—C8—C9	60.3 (2)	C24—C23—C25—C22^ii^	−179.3 (3)
C7—C8—C9—C10	−77.14 (19)	C25—C23—C24—C22	−179.3 (3)
C8—C9—C10—C11	151.51 (14)	C26^iii^—C26—C27^iii^—C27	0 (12936568)
C8—C9—C10—C15	−29.5 (2)	C26^iii^—C26—C27^iii^—C28	−179.6 (4)
C9—C10—C11—C12	179.26 (13)	C27^iii^—C26—C26^iii^—C28^iii^	−179.6 (5)
C9—C10—C11—C19	−5.1 (3)	C27^iii^—C26—C26^iii^—C29^iii^	−0.2 (4)
C9—C10—C15—C14	173.81 (13)	C26^iii^—C26—C28—C27^iii^	0.30 (17)
C11—C10—C15—C14	−7.2 (3)	C28—C26—C26^iii^—C27	179.6 (4)
C15—C10—C11—C12	0.2 (2)	C28—C26—C26^iii^—C29^iii^	−0.6 (4)
C15—C10—C11—C19	175.82 (12)	C26^iii^—C26—C29—C27	−0.16 (17)
C10—C11—C12—C13	6.5 (3)	C29—C26—C26^iii^—C27	0.2 (3)
C10—C11—C19—C21^i^	−76.48 (16)	C29—C26—C26^iii^—C28^iii^	0.6 (4)
C12—C11—C19—C21^i^	99.13 (14)	C28—C26—C27^iii^—C27	179.6 (3)
C19—C11—C12—C13	−169.30 (12)	C28—C26—C29—C27	−179.5 (4)
C11—C12—C13—C14	−6.3 (3)	C29—C26—C28—C27^iii^	179.6 (4)
C11—C12—C13—C20	173.34 (13)	C26^iii^—C27—C27^iii^—C28	−179.6 (5)
C12—C13—C14—C15	−0.6 (2)	C26^iii^—C27—C27^iii^—C29^iii^	0.2 (4)
C12—C13—C14—C16	179.13 (12)	C26^iii^—C27—C28^iii^—N3^iii^	−178.8 (6)
C12—C13—C20—C21	−24.0 (2)	C27^iii^—C27—C28^iii^—N3^iii^	−178.5 (5)
C14—C13—C20—C21	155.60 (13)	C27^iii^—C27—C28^iii^—C26^iii^	0.3 (2)
C20—C13—C14—C15	179.77 (12)	C28^iii^—C27—C27^iii^—C26	179.6 (5)
C20—C13—C14—C16	−0.5 (3)	C28^iii^—C27—C27^iii^—C29^iii^	−0.2 (5)
C13—C14—C15—C10	7.4 (3)	C27^iii^—C27—C29—N4	−177.8 (5)
C13—C14—C16—C17	−78.71 (17)	C27^iii^—C27—C29—C26	0.2 (2)
C15—C14—C16—C17	101.02 (14)	C29—C27—C27^iii^—C26	−0.2 (3)
C16—C14—C15—C10	−172.37 (13)	C29—C27—C27^iii^—C28	0.2 (5)
C14—C16—C17—C18	−75.03 (13)	C28^iii^—C27—C29—N4	2.4 (9)
C16—C17—C18—C2	69.70 (16)	C28^iii^—C27—C29—C26	−179.6 (6)
C11—C19—C21^i^—C20^i^	−122.71 (10)	C29—C27—C28^iii^—N3^iii^	1.3 (9)
C13—C20—C21—C19^i^	−62.02 (16)	C29—C27—C28^iii^—C26^iii^	−179.9 (6)

Symmetry codes: (i) −*x*, −*y*, −*z*; (ii) −*x*, −*y*+1, −*z*+1; (iii) −*x*+1, −*y*, −*z*; (iv) *x*, *y*+1, *z*; (v) *x*−1, *y*, *z*; (vi) −*x*, −*y*+1, −*z*; (vii) *x*+1, *y*, *z*; (viii) −*x*+1, −*y*, −*z*+1; (ix) *x*, *y*−1, *z*; (x) −*x*+1, −*y*+1, −*z*+1; (xi) −*x*+1, −*y*+1, −*z*; (xii) *x*+1, *y*+1, *z*; (xiii) −*x*, −*y*, −*z*+1; (xiv) *x*, *y*, *z*+1; (xv) *x*, *y*, *z*−1; (xvi) *x*−1, *y*−1, *z*.
